# Comparing the efficacy of botulinum toxin with tizanidine in upper limb post stroke spasticity

**Published:** 2013

**Authors:** Mohammad Yazdchi, Zahra Ghasemi, Hanieh Moshayedi, Reza Rikhtegar, Somayeh Mostafayi, Hale Mikailee, Safa Najmi

**Affiliations:** 1 Associate Professor, Department of Neurology, Tabriz University of Medical Sciences, Tabriz, Iran; 2 Resident, Department of Neurology, Tabriz University of Medical Sciences, Tabriz, Iran; 3 Associate Professor, Department of Internal Medicine, Tabriz University of Medical Sciences, Tabriz, Iran; 4 Assistant Professor, Department of Neurology, Tabriz University of Medical Sciences, Tabriz, Iran

**Keywords:** Tizanidine, Dysport, Modified Ashworth Scores, Action Research Arm Test, Post-Stroke Upper Limb Spasticity

## Abstract

**Background:**

This study evaluated the efficacy of focal intramuscular injection of botulinum (BoNT) toxin type A in comparison with oral tizanidine (TZD) in treatment of post-stroke upper limb spasticity.

**Methods:**

This was a double-blinded randomized clinical trial that recruited 68 patients with post-stroke upper limb spasticity. Thirty-four patients received BoNT (Dysport^®^) injections in affected muscles of upper limb at the baseline and week 12. Thirty-four patients were treated with tizanidine (Sirdalude) by gradual increase in dosage of 2mg/week to reach maximum 24mg at week 12. Modified Ashworth Scale (MAS) and Action Research Arm Test (ARAT) were evaluated at the baseline, week 12 and week 24 for all the participants.

**Results:**

The mean score of MAS reduced from 3.32 and 3.13 at baseline to 1.79 and 1.56 at week 24 on elbow and wrist joints, respectively (P < 0.01). However, there were only reductions from 2.79 and 2.77 to 2.32 and 2.31 (P < 0.001) in TZD group. ARAT increased from 1.79 to 10.97 (P < 0.001) in BoNT group. ARAT increased from 11.08 to 11.35 in TZD group (P = 0.026).

**Conclusion:**

BoNT injection was safe and effective in reducing post-stroke upper extremity spasticity in comparison with TZD.

## Introduction

Stroke is the main cause of disability all around the world. Approximately 40% of the stroke survivors never acquire their adequate limb function.^1–3^

One of the causes of disability in post-stroke is spasticity with the frequency of 41.6 as mentioned in a recent multicenter study in Thailand.^4^

Spasticity is an increased resistance impassive movement at a joint that depends upon velocity of an action. It can interfere with positioning of limb toward body, implementing daily routines and self-care.^5^

Although a large number of oral antispastic agents are available in addition with physiotherapy in treatment of spasticity, systemic adverse effects and inadequate efficacy of these drugs are limited factors in their administration.^6, 7^

The most common oral antispastic agents are diazepam, dantrolene, baclofen, clonidine, tizanidine, and gabapentin. All of them may cause dizziness, confusion, sedation, hypotension and other central or systemic side effects^5^ due to their systemic distribution and non-selective mechanisms of action.

Botulinum toxin type A (BoNT/A) blocks neurotransmitter release at neuromuscular junction, and therefore induces muscle paralysis that resulted in reduction of muscle tone. Therefore, BoNT does not directly improve function, but allows easier movement to occur at joints by reducing spasticity. Moreover, because BoNT is used focally, it does not induce any systemic side-effects.

In many recent studies, although the safety and efficacy of focal intramuscular injection of BoNT has been demonstrated in treatment of upper limb spasticity due to stroke,^8–12^ some physicians still prefer administering oral antispastic agents without considering systemic adverse effects of these medications toward BoNT.

The present study was done to evaluate the efficacy of focal intramuscular injection of botulinum toxin type A (BoNT) in comparison with oral tizanidinein (TZD) in treatment of post-stroke upper extremity's spasticity.

## Materials and Methods

This was a double-blinded randomized clinical trial. Based on previous studies in which reduction of spasticity was their main objective, the sample size calculated for 80% power to reach anticipated benefit of 20% between the two study groups. Sixty-eight patients were recruited to the study with significance level of 5% (P < 0.05).

Since July 2010 to December 2012, 68 eligible patients with our inclusive and exclusive criteria were recruited (that are mentioned below thoroughly) and came to follow up visits to Imam Reza University Hospital and Neurology Clinic and were randomly allocated into two equal groups in which were treated with either TZD or BoNT for their upper limb spasticity. Informed consent was obtained from all of them for participation in the study. This study was performed according to the Declaration of Helsinki and was approved by the local Ethics Committee of Tabriz University of Medical Sciences.

Patients older than 35 years who had experienced stroke (ischemic or hemorrhagic that documented by computed tomography or magnetic resonance imaging) with onset of at least 3 months ago, were evaluated by Modified Ashworth Scale (MAS) for their upper limb spasticity. And patients with minimum score of 2 on the MAS were included. Patients who suffered from severe dementia or impaired consciousness were excluded from the study. In addition, patients who received BoNT injection into affected muscles in at least 3 months before recruitment and those who were older than 70 years old were excluded.

Eligible patients went through the baseline assessment of MAS^13^ and ARAT^14^ for their evaluation of severity for spasticity and function of affected upper limb by a neurology resident, respectively.

Then, patients were randomly assigned into two groups in which treated by either BoNT or TZD. They were evaluated by MAS and ARAT or their spasticity and function of upper extremities by the same neurology resident who was blinded for the mode of therapy in a regular period of 12 weeks and 24 weeks after initiation.

All the patients offered to have rehabilitative treatments with the same program at the same university physical therapy center. The physiotherapy program consisted of 45-60 min of strengthening, stretching and passive range of motion exercises, electrical stimulation and endurance exercise three times a week throughout the study.

In BoNT group, patients received injections into dominant spastic muscles of the upper extremities according to the same neurologist at baseline and week 12.

In this study, Dysport 500U including clostridium botulinum type A and toxin-hemagglutinin complex, IPSEN Ltd were used and each vial diluted with 2.5ml sodium chloride 0.9%.

Approximately, biceps brachii (150-200U), flexor carpi radialis (50-100U), flexor carpi ulnaris (50-100U) and flexor digitorum profundus (100-150U) were the most common injected muscles, respectively in all the patients. The maximum dosage of 1000U was limitation point in each time for an upper extremity.

In TZN group, patients were administrated with Sirdalude (Novartis) with initiated dosage of 2mg and gradual increase of 2 mg weekly to reach 24 mg at week 12 and continued the same dosage of 24 mg to week 24 to the end of the study.

All the patients were informed about the possible frequent adverse effects of BoNT (muscle weakness, injection site pain or hemorrhage) or TZN (sedation, dry mouth, abdominal pain, nausea and vomiting, dizziness, hypotension) according to their treatment type. Besides, the neurologist contact number was provided and they were asked to inform the neurologist as soon as possible in the case of any occurrence of adverse effects.

The results were analyzed through descriptive statistical methods Wilcoxon signed-ranks test, Mann-Whitney test and SPSS for Windows 19 (SPSS Inc., Chicago, IL, USA) were used as the statistical tests. P value less than 0.05 was considered as a significant level.

## Results

Thirty-four patients were enrolled in each treatment group who were justified for gender and age. There were 21 men and 13 women with mean age of 67.5 (35-70) years old in BoNT group and there were 18 men and 16 women with mean age of 64.7 (51-68) years old in TZD group.

The mean scores of MAS showed that BoNT has reduced muscle tone at elbow and wrist joints from 3.32 and 3.13 at baseline to 1.79 and 1.56 at week 24, respectively (P < 0.01) (Table 1).

**Table 1:** The clinical characteristics of the patients

	BoNT Baseline, week 12, week 24	TZD Baseline, week 12, week 24
MAS: Mean (SD)		
Elbow joint	3.32 (0.63), 2.17 (0.45), 1.79 (0.47)	2.79 (0.41), 2.67 (0.47), 2.32 (0.58)
Wrist joint	3.13 (0.59), 2.2 (0.43), 1.56 (0.44)	2.77 (0.40), 2.65 (0.45), 2.31 (0.54)
ARAT: Mean (SD)	1.79 (3.38), 5.64 (3.8),10.79 (4.57)	11.02 (5.45), 11.08 (5.51), 11.35 (5.85)

MAS: Modified Ashworth Scale; ARAT: Action Research Arm Test

However, figures at TZD group showed the reduction of mean scores from 2.79 and 2.77 to 2.32 and 2.31 from baseline to week 24 at joints elbow and wrist, respectively (Table 1).

Regarding the figures, we came to the conclusion that BoNT improved intensity of spasticity in upper extremity much more than TZD (-1.57 vs. -0.47) at elbow and (-1.53 vs. -046) at wrist joints, even though P-values were statistically significant at both (Table 1).

The mean score of ARAT at baseline for BoNT and TZD were 1.79 and 11.02, respectively. At week 12 and 24, the figures increased significantly at BoNT group to 5.64 and 10.97 (P < 0.01). However, in TZD group, the mean scores were increased only to 11.08 at week 12 and 11.35 at week 24 (Table 1).

Although, P-value was statistically significant compared with ARAT scores before and after TZD (P = 0.026), it was obvious that BoNT made much more significant improvement on ARAT and subsequently in function of upper limb.

All of these findings are summarized in Table 1.

No statistical analysis was done for adverse effects, even though 20 patients ended up in side effects of TZD and quitted study. Other eligible participants were replaced to prevent reduction and sample loss in sample size.

Seven patients could not tolerate the dosage of 12 mg and 13 out of 20 discontinued receiving TZD when the dosage reached to 24 mg. Sedation and dizziness were the main causes of adverse effects in 17 patients. Besides, three patients could not continue receiving TZD due to abdominal pain and nausea. No adverse effect was found at BoNT group. This showed that BoNT was safe at the used dosages of this study.

## Discussion

Our study statistically demonstrated that BoNT and TZD significantly reduced muscle tone. However, it seems that focal injections of BoNT were more effective than TZD to improve in spasticity at elbow and wrist joints.

Even though ARAT scores showed a significant increase in BoNT group, BoNT did not seem to improve active function or a significant change in quality of life.

There was only one study that compared the effectiveness of BoNT and TZD in treatment of spasticity in upper limb. Our study results were similar to Simpson et al. who demonstrated focal injections of BoNT improved spasticity more than TZD or placebo.^15^ However, our results were not in accordance with this study which stated TZD was not effective in treatment of post-stroke upper extremity's spastisity.^15^

This fact was due to failure of reaching the therapeutic dosage of TZD caused by occurrence of adverse effects at the study of Simpson et al.^15^

Whereas Gelber et al. in a study with 47 stroke patients showed TZD with mean dosage of 20mg/day reduced muscle tone significantly.^16^

In addition, in a randomized, double-blinded, placebo controlled clinical trial, Meythaler et al.^18^ showed that TZD with maximum dosage of 36mg/day for 8 weeks in 17 patients with acquired brain injury (8 TBI, 9 strokes) reduced spasticity.^17^

Our study was in accordance with Gelber et al. that TZD was effective as an antispastic agent.

Our study showed improvement of ARAT score by injections of BoNT; however, it was in opposition with Cousins et al.^18^ that demonstrated low dosage of BoNT could not result in adequate arm function that was measured by ARAT. This might be because they failed to use enough dosage of BoNT in appropriate muscles.

## Conclusion

In keeping with previous studies^19–21^, our study showed focal intramuscular injections of BoNT was safe and more effective in reduction of spasticity and improvement in function of post-stroke upper extremity spasticity. Finally, it seems that BoNT was more effective and safer compared with TZD.
